# Meta-Analysis of QTL Mapping and GWAS Reveal Candidate Genes for Heat Tolerance in Small Yellow Croaker, *Larimichthys polyactis*

**DOI:** 10.3390/ijms26041638

**Published:** 2025-02-14

**Authors:** Feng Liu, Haowen Liu, Tianle Zhang, Dandan Guo, Wei Zhan, Ting Ye, Bao Lou

**Affiliations:** 1 State Key Laboratory for Managing Biotic and Chemical Threats to the Quality and Safety of Agro-Products, Institute of Hydrobiology, Zhejiang Academy of Agricultural Sciences, Hangzhou 310021, China; 18252485520@163.com (T.Z.); hmguodandan@163.com (D.G.); weizhan1113@163.com (W.Z.); 15tye@stu.edu.cn (T.Y.); 2 Zhejiang Key Laboratory of Coastal Biological Germplasm Resources Conservation and Utilization, Wenzhou 325005, China; 3 College of Life Sciences, China Jiliang University, Hangzhou 310018, China; liuhaowendandan@163.com

**Keywords:** *Larimichthys polyactis*, heat tolerance, genetic linkage map, QTL, GWAS

## Abstract

High temperatures present considerable challenges to global fish growth and production, yet the genetic basis of heat tolerance remains underexplored. This study combines quantitative trait locus (QTL) mapping and genome-wide association studies (GWAS) to examine the genetics of heat tolerance in *Larimichthys polyactis*. As a result, a genetic linkage map was constructed with 3237 bin markers spanning 24 linkage groups and totaling 1900.84 centimorgans, using genotyping-by-sequencing of a full-sib family comprising 120 progeny and their two parents. Based on this genetic linkage map, QTL mapping identified four QTLs associated with heat tolerance, which encompassed 18 single nucleotide polymorphisms and harbored 648 genes within the QTL intervals. The GWAS further disclosed 76 candidate genes related to heat tolerance, 56 of which overlapped with the QTL results. Enrichment analysis indicated that these genes are involved in immune response, development, lipid metabolism, and endocrine regulation. qPCR validation of 14 prioritized genes, which were simultaneously enriched in Gene Ontology and Kyoto Encyclopedia of Genes and Genomes pathways, confirmed significant upregulation of *smpd5*, *polr3d*, *rab11fip2*, and *gfpt1*, along with downregulation of *gpat4* and *grk5* after 6 h of heat stress. These findings demonstrate their responsiveness to elevated high temperatures. This meta-analysis of QTL mapping and GWAS has successfully identified functional genes related to heat tolerance, enhancing understanding of the genetic architecture underlying this critical trait in *L. polyactis*. It also provides a molecular breeding tool to improve genetic traits associated with heat tolerance in cultured *L. polyactis*.

## 1. Introduction

Under the context of global climate change, extreme weather events, especially heatwaves, have become increasingly frequent and intense [1]. This intensification trend poses a considerable challenge to aquaculture production, as extremely high temperatures exert extensive stress on farmed fish, potentially even threatening their survival [2]. Such stress can lead to substantial and irreversible damage to fish tissues, thereby triggering physiological and metabolic changes that adversely affect both the growth and development of fish [3,4]. The small yellow croaker, *Larimichthys polyactis*, is a marine demersal oceanodromous fish that belongs to the Sciaenidae family within the order Perciformes. It predominantly inhabits the western shores of South Korea, as well as the Bohai Sea and East China Sea [5]. Highly valued for its exceptional nutritional content and superior meat quality, it is a highly sought-after and economically important marine species in both China and Korea. Unfortunately, wild populations have experienced severe depletion due to overfishing and habitat degradation [6]. In response to the increasing demand for this fish, marine farming initiatives have been launched in both China and Korea as a sustainable alternative to fishing [7,8]. However, prolonged high temperatures during summer trigger a pronounced stress response in *L. polyactis*, leading to a higher incidence of diseases and substantial economic losses for the aquaculture industry. Thus, to develop heat-tolerant strains through genomic selection, it is essential to uncover the genetic basis of heat tolerance in *L. polyactis* as well as the complex molecular mechanisms that regulate its response to heat stress. Genomic selection has substantially accelerated the genetic breeding of traits that are challenging to measure directly, such as disease resistance, environmental tolerance, and meat quality [9].

A critical prerequisite for employing genomic selection is the screening and identification of loci or genes associated with the target trait. Quantitative trait loci (QTL) mapping has emerged as an effective tool for identifying trait-related loci and genes, and it has been widely applied across various fish species. A wealth of data regarding QTL and genetic bases related to heat stress has been published for several marine fish, including Chinook salmon (*Oncorhynchus tshawytscha*) [10], turbot (*Scophthalmus maximus*) [11], and rainbow trout (*Oncorhynchus mykiss*) [12]. Constructing high-quality genetic linkage maps by using a large number of molecular markers is a fundamental step for accurate QTL localization. With advancements in next-generation sequencing technology and genotyping-by-sequencing (GBS) approaches, the use of single-nucleotide polymorphism (SNP) markers has increasingly been prioritized for map construction because of their abundance, widespread availability, and ease of genotyping [13]. High-density genetic linkage maps are invaluable tools for the genetic breeding of economically important species, supporting genome assembly, fine-scale QTL mapping, and positional cloning of target genes [10,14]. Additionally, genome-wide association studies (GWAS) serve as another critical tool for identifying functional loci and discovering genes. GWAS plays a vital role in elucidating the genetic mechanisms underlying important traits, including growth rate [15], disease resistance [16], temperature tolerance [17], salinity tolerance [18], and ammonia tolerance [19] in aquatic animals.

QTL mapping and GWAS have been extensively used to unravel the genetic basis of complex quantitative traits. While QTL mapping has its limitations, these can be compensated for by GWAS, which enables a more precise identification of the candidate gene region [20]. However, GWAS may also exhibit a high false positive error rate because of influences of population structure and size [21]. Therefore, integrating both mapping strategies offers a powerful and complementary approach to genetic mapping, effectively overcoming each method’s limitations when applied independently. This combined strategy has been successfully implemented in species such as *Crassostrea gigas* [22], *Triticum aestivum* [23], and *Zea mays* [24] to identify key genes associated with economically important traits. However, to date, studies on QTL mapping and GWAS related to temperature tolerance in *L. polyactis* remain limited. In this study, an integrated analysis was conducted using QTL mapping and GWAS to identify key genes associated with heat tolerance in *L. polyactis*.

## 2. Results

### 2.1. Statistical Analysis of Heat-Tolerant Phenotypes

**Figure 1:**
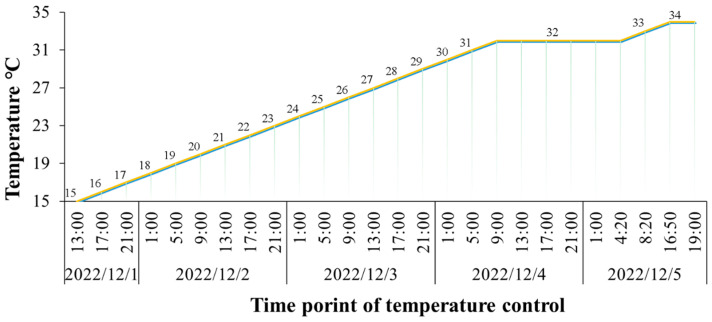
Temperature changes at each time point during the heat stress experiment. Temperature increased from 15 °C to 32 °C by 1 °C every 4 h. Subsequently, if no mortality occurred for four consecutive hours at this temperature, an additional 1 °C increase was implemented, and this process continued until all fish had succumbed.

The temperature control protocol systematically increased the temperature by 1 °C every 4 h, ultimately achieving a maximum of 32 °C. Subsequently, if no mortality was observed for four consecutive hours at this temperature, an additional 1 °C increase was implemented, and this process continued until all fish had succumbed. The procedure is illustrated in Figure 1. During the experiment, 120 fish from a full-sib family were tested. The first fish died when the water temperature reached 31 °C, marking the beginning of significant heat stress. The specific time at which this critical temperature was reached, designated as T0, was documented as 64 h (T0 = 64 h).

The survival durations of each individual were meticulously recorded throughout the experimental period. After 1400 min of heat stress application, no further mortalities were recorded for a consecutive 4 h. Based on this observation, fish that died prior to this 1400 min threshold were assigned a code of 0, whereas those that survived beyond this point were coded as 1. These codes served as indicators of survival status and are detailed in Table S1.

**Figure 2:**
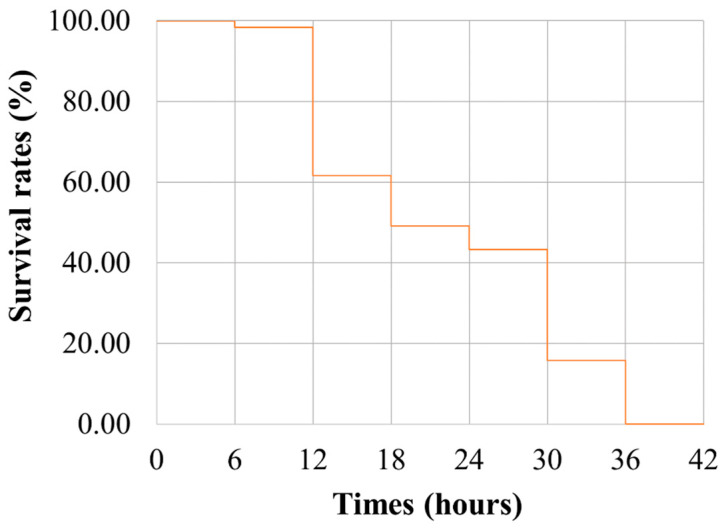
Survival curve of *Larimichthys polyactis* during heat stress. Cumulative mortality reached nearly 50% after 18 h of heat stress. The heat stress experiment lasted for 36 h, with the first fish succumbing to stress after only 190 min. The average survival duration was calculated to be 19.72 h, with peak mortality occurring between the 12th and 30th hours of the heat stress exposure.

Figure 2 illustrates the survival curve derived from the survival duration data. The curve shows that cumulative mortality reached nearly 50% after 18 h of heat stress. The heat stress experiment lasted for 36 h, with the first fish succumbing to stress after just 3.2 h (190 min). An analysis of the phenotypic data statistics (Table S2) demonstrated significant fold changes in the survival duration phenotype among fish. Specifically, the average survival duration was calculated to be 19.72 h, with peak mortality occurring between the 12th and 30th h of heat stress exposure.

### 2.2. Sequencing and Genotyping

A total of 9.02 × 10^11^ bp of clean data were obtained from the two parents and their 120 progenies following high-throughput sequencing. After filtering, the male parent provided 8.60 × 10^9^ bp of clean data, while the female parent provided 8.26 × 10^9^ bp. The clean data of progenies ranged from 6.14 × 10^9^ to 9.04 × 10^9^ bp. The average Q20 and Q30 scores exceeded 96.5% and 91.2%, respectively, with guanine–cytosine (GC) content ranging from 41.60% to 42.87%. The clean data were deposited in the Sequence Read Archive (http://www.ncbi.nlm.nih.gov/sra, accessed on 9 February 2025) of the NCBI under GenBank accession number PRJNA1184984. Furthermore, the average mapping rate across all samples exceeded 98.86% (Table S3), leading to the identification of 7,479,427 SNPs (Table S4).

### 2.3. Construction of Linkage Maps

**Figure 3:**
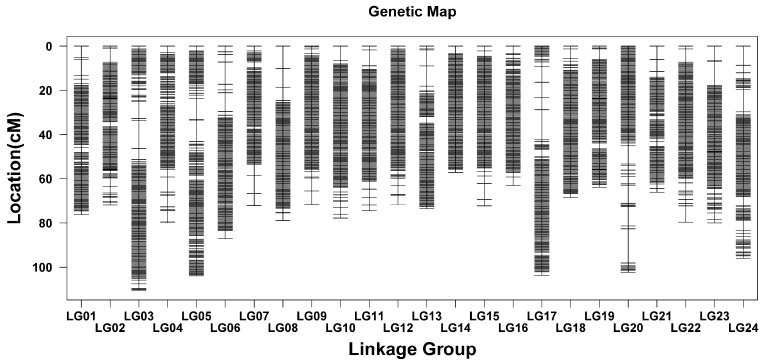
Length and marker distribution of 24 linkage groups in the sex-averaged bin map. The ordinate indicates the genetic distance.

After genotyping and filtering of markers, a refined set of 3,967,902 SNPs was obtained. Among these, 3,958,329 markers were used to construct the sex-averaged linkage map, while 9573 high-quality markers were discarded or not located. The resulting linkage map measured 1900.85 cM, with an average marker interval of 0.0001 cM. The linkage map comprised 24 linkage groups (LG) aligned with the haploid chromosome number (Table 1). The sex-averaged bin maps featured 3237 bin markers, with an average span of 0.59 cM. Both the length and number of LGs in the bin map were identical to those in the linkage map. The genetic length of LGs ranged from 57.10 cM (LG14) to 110.40 cM (LG03), averaging 79.2 cM. The average interval length of LGs varied from 0.44 cM (LG14) to 0.81 cM (LG20), with a mean value of 0.59 cM (Figure 3; Table S5).

### 2.4. QTL Mapping and Identification of Heat Tolerance Candidate Genes

**Figure 4:**
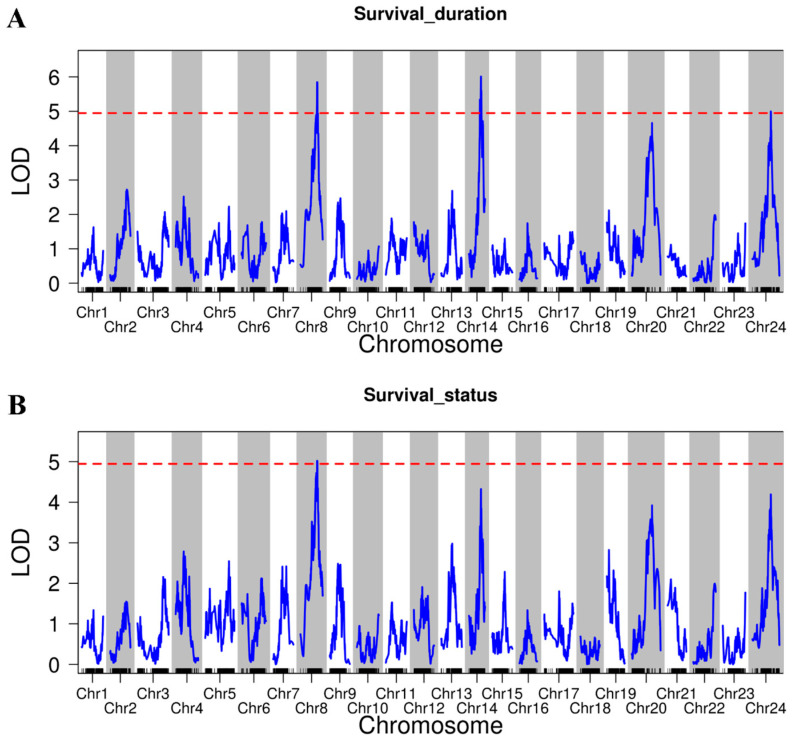
QTL analysis for heat tolerance traits in the full-sib family using SNP data of *L. polyactis*. (**A**) Survival duration under high-temperature stress. Three major genome-wide significant QTL intervals were identified with LOD values of 5.85, 6.01, and 5.00, with percentages of explained variance of 10.13%, 13.08%, and 6.98%, respectively. (**B**) Survival status under high-temperature stress. One major genome-wide significant QTL interval was identified with a LOD value of 5.02 and a percentage of explained variance of 17.52%. The horizontal axis indicates linkage group numbers. The vertical axis represents LOD values. The red line indicates the determined LOD (=4.95).

To gain a deeper understanding of the heat tolerance in *L. polyactis*, the QTLs associated with heat tolerance traits (namely survival duration and survival status) were analyzed. Using the threshold value of LOD = 4.95 (*p* < 0.05), a total of four QTLs encompassing 20 SNPs linked to heat tolerance traits were identified (Table S6). The LOD values for these QTLs ranged from 5.00 to 6.01. For the trait of survival duration, three QTLs were discovered across LG8, LG14, and LG24. These QTLs encompassed 18 SNPs and explained phenotypic variances of 10.13%, 13.08%, and 6.98%, respectively. For the trait of survival status, a single QTL, comprising two SNPs, was identified on LG8, which accounted for a phenotypic variance of 17.52%. It is worth noting that the significant QTLs for survival status overlapped with one of the QTLs for survival duration (Figure 4; Table 2). This overlap indicates that genes located within the shared QTL interval may play a pivotal role in determining the heat tolerance of *L. polyactis*.

To further examine these potential genes, a total of 616 genes within the QTL interval for survival duration and 375 genes within the QTL interval for survival status were identified. Intriguingly, all 343 genes located within the QTL interval for survival duration on LG8 were also present among the 375 genes identified within the QTL interval for survival status. The names and annotation information for these genes are provided in Table S6.

### 2.5. GWAS Analysis of Heat Tolerance

**Figure 5:**
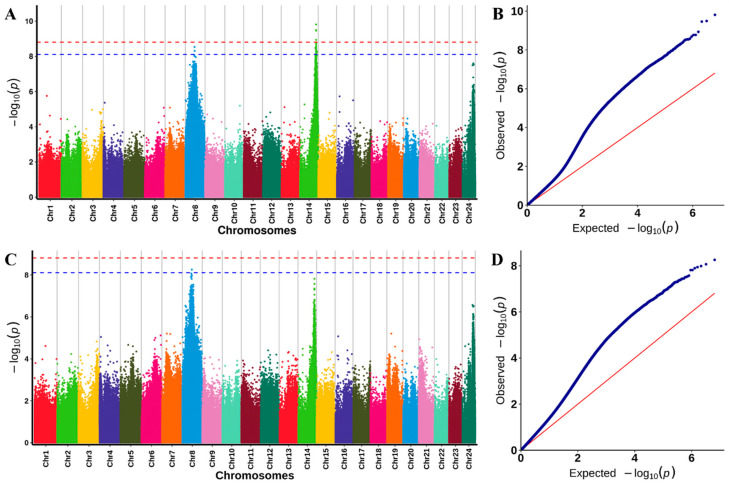
GWAS analysis of resistance to high temperature in *L. polyactis*: (**A**) Manhattan plot for GWAS of survival duration; (**B**) Q-Q plot of survival duration; (**C**) Manhattan plot for GWAS of survival status; (**D**) Q-Q plot of survival status. The red and blue dashed lines indicate a genome-level suggestive significance threshold *p* value of 7.77 × 10^−9^. The red lines in B and D represent the expected distribution of −log_10_ (*p*) values under the null hypothesis, while deviations from this line (blue) reflect the *p*-value from which the regression of the phenotype on the SNP deviates from what would be expected by chance.

To validate the detected QTLs, GWAS was used to identify SNPs associated with survival duration and survival status (Table S7). After applying the SNP filtering process described in the methods, a total of 6,435,495 SNPs were selected for further analysis. Using a Bonferroni-corrected significance threshold of 7.77 × 10^−9^ (0.05/6,435,495), 27 SNPs were found to be significantly associated with survival duration, while 1 SNP was significantly associated with survival status (Figure 5; Table 3).

Utilizing the MAGMA tool and referencing the genome of *L. polyactis*, both downstream and upstream gene searches were conducted to identify potential candidate genes. As a result, 76 candidate genes putatively related to heat resistance in *L. polyactis* were identified (Table 3). Among these, 66 genes were found to be associated with survival duration, while 10 genes were linked to survival status. It is worth noting that all 10 genes related to survival status were also included within the set of genes associated with survival duration, suggesting considerable overlap. Therefore, a total of 66 genes were ultimately identified as being related to heat tolerance. The names and their corresponding annotation information of genes are provided in Table S8.

### 2.6. Functional Enrichment Analysis of Candidate Genes

Upon comparing the candidate genes obtained through QTL mapping and GWAS, 56 genes were found to be common in both methods (Table S9). These common genes were subsequently subjected to enrichment analysis, which showed that 40 genes had associated GO terms and 16 genes were associated with KEGG pathways. The GO enrichment test was performed by comparing the candidate list of 40 genes with their associated GO terms to a reference list of 15,499 genes with associated GO terms. The results indicated a total of 50 GO terms were significantly enriched in the candidate gene list, as compared to the reference list, at a significance level of *p* = 0.05.

**Figure 6:**
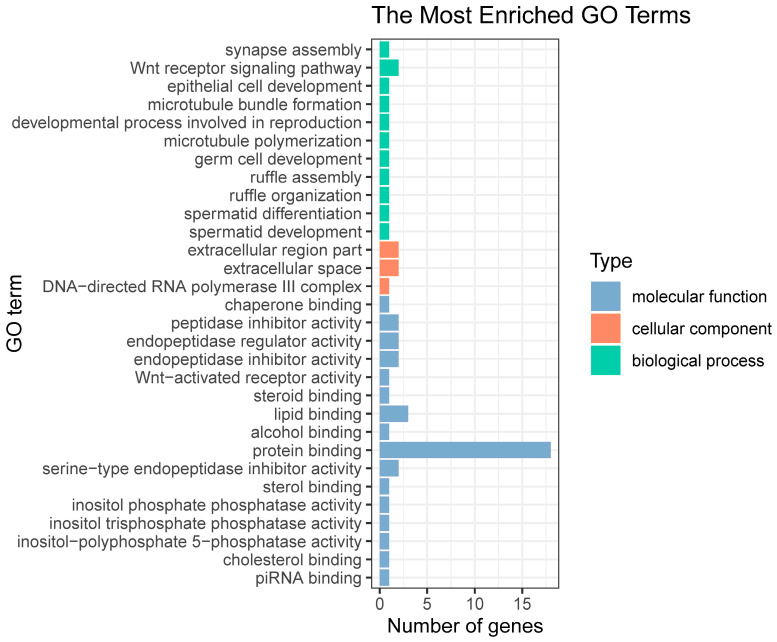
GO enrichment analysis of overlap genes identified by QTL and GWAS for heat tolerance trait in *L. polyactis*. Genes were annotated in three categories: biological process, cellular component, and molecular function. Top 20 terms of GO enrichment analysis. The Y-axis represents the name of each GO term, and the X-axis represents the number of genes in each term.

**Figure 7:**
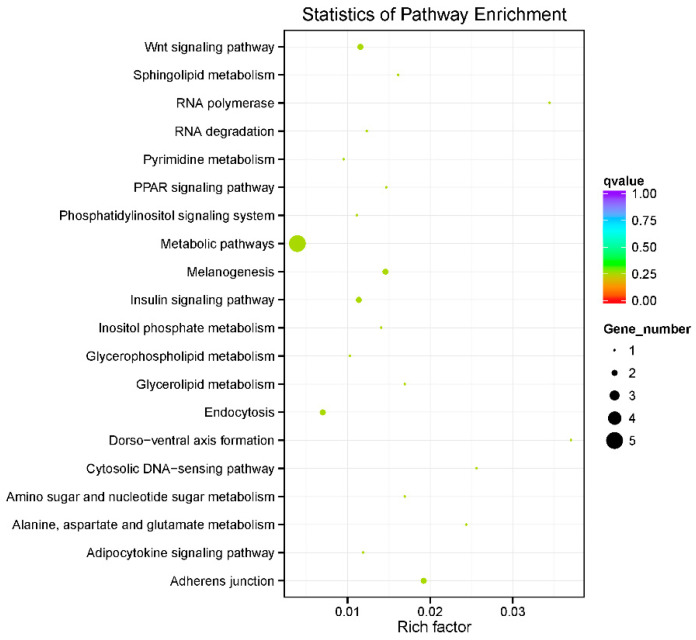
KEGG enrichment analysis of gene overlap identified by QTL and GWAS for heat tolerance trait in *L. polyactis*. All 21 enriched pathways are shown, and the most enriched pathways include the Adherens junction, melanogenesis, Wnt signaling pathway, insulin signaling pathway, Endocytosis, and metabolic pathways. Among these pathways, the two pathways of Adherens junction and melanogenesis were significantly enriched. The pathways were ranked according to their statistical significance.

The enriched GO terms indicated that the functions of these genes are related to reproduction and development, enzyme activity, nervous system function, and tissue organization (Figure 6; Table S10). These findings offer valuable insights into the biological processes associated with heat tolerance in *L. polyactis*. For instance, enrichment of GO terms such as “Wnt receptor signaling pathway” and “Wnt-activated receptor activity” implies that responses to stimuli are initiated by high-temperature exposure. Analogously, KEGG pathways such as the “cytosolic DNA-sensing pathway” (acting as a pattern recognition receptor) and key immune signal transduction pathways (including the “Wnt signaling pathway” and “phosphatidylinositol signaling system”) were enriched. These enrichments indicate the involvement of the immune system in the response to heat stress (Figure 7; Table S11).

Moreover, GO terms related to “spermatid development”, “spermatid differentiation”, “germ cell development”, and “developmental processes involved in reproduction”, as well as the KEGG pathway for “development and regeneration”, were significantly enriched. These findings suggest that heat stress may affect the reproduction of this species of fish. Furthermore, the enrichment of GO terms such as “cholesterol binding”, “sterol binding”, and “steroid binding”, coupled with KEGG pathways including “glycerolipid metabolism”, “sphingolipid metabolism”, and “glycerophospholipid metabolism”, suggests the involvement of lipid metabolism during heat stress. Notably, enriched KEGG pathways for “melanogenesis”, “insulin signaling pathway”, “PPAR signaling pathway”, and “adipocytokine signaling pathway” highlight the role of endocrine system regulation in response to heat stress (Figure 6 and Figure 7).

### 2.7. Identification of High-Temperature Response Gene Candidates

**Figure 8:**
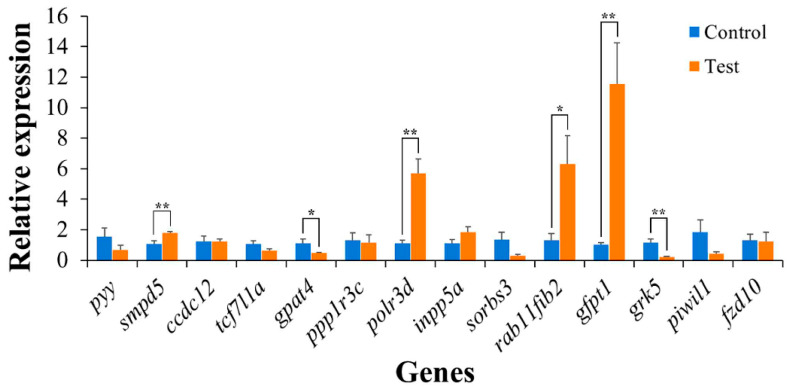
Expression of candidate genes subjected to heat stress. The fish in the test group were subjected to a temperature of 32 °C for 6 h, and the fish in the control group were cultured in the normal water temperature of 20 °C. After heat stress for 6 h, six candidate genes were significantly differently expressed; *smpd5*, *polr3d*, *rab11fip2*, and *gfpt1* were significantly upregulated, while *gpat4* and *grk5* were significantly downregulated. * indicates a significant difference (*p* ≤ 0.05); ** indicates a highly significant difference (*p* ≤ 0.01).

To validate the results predicted in this study, qPCR analysis was conducted to further investigate the expression patterns of 14 genes related to heat tolerance that were simultaneously enriched in GO and KEGG pathways. The expression levels of six candidate genes exhibited significant alterations in response to the high-temperature challenge (Table 4; Figure 8). Specifically, after 6 h of heat stress, the expressions of *smpd5*, *rab11fip2*, *polr3d*, and *gfpt1* genes were significantly upregulated by approximately 1.64-fold, 4.82-fold, 5.13-fold, and 11.22-fold, respectively. Conversely, the expression of *gpat4* and *grk5* genes was downregulated by approximately 2.40-fold and 5.24-fold, respectively, at the same time point (Figure 8). These findings suggest that these candidate genes are responsive to high-temperature exposure, thereby confirming the predicted results.

## 3. Discussion

In the context of global climate change and expanding aquaculture areas, heat stress has emerged as a formidable challenge confronting fish populations [25]. Consequently, cultivating heat-tolerant fish varieties is imperative for the advancement of the aquaculture industry. However, genetically enhancing temperature tolerance in fish with traditional methods presents a challenge because of measurement inaccuracies and the often low heritability of this trait [26,27]. Thus, the adoption of advanced molecular breeding technologies is indispensable to address this issue.

To unravel the genetic regulatory mechanisms underpinning heat tolerance, it is essential to explore genes associated with this trait. Several economically important fish species, including rainbow trout (*Oncorhynchus mykiss*) [28], common carp (*Cyprinus carpio* L.) [29], Arctic charr (*Salvelinus alpinus*) [30], and turbot (*Scophthalmus maximus*) [11], have been studied to elucidate the genetic basis of their temperature tolerance. Recently, transcriptomic and proteomic approaches have been employed to examine the effects of heat stress on *L. polyactis*, and the results have offered valuable insights into potential pathways as well as a suite of genes and proteins associated with heat stress [31,32]. Despite the intricacies involved in studying stress tolerance, which is regulated by quantitative traits encompassing multiple biochemical and physiological processes [33], identifying key candidate genes that confer heat tolerance remains crucial.

QTL mapping and GWAS are powerful techniques for deciphering the genetic architecture underlying quantitative traits [34,35]. Fueled by the surge in high-quality genomic resources, these two methods have substantially contributed to understanding the genetic architecture and mechanisms of traits of interest [9]. A crucial determinant of the accuracy of QTL mapping and GWAS is the precision of phenotypic data. As is well known, when test subjects are exposed to acute temperature increases, individuals die too rapidly, making it difficult to distinguish survival duration phenotypes among deceased individuals. Conversely, chronic temperature increases often prolong the experimental duration, thereby making it susceptible to disturbances from various factors, including diseases and nutritional status. To address these issues, this study innovatively introduces a method where full-sib family individuals are subjected to high-temperature stress by gradually increasing the water temperature to 32 °C at a relatively slow rate of 4 °C per hour. This approach allows for precise documentation of the survival duration of each individual under high-temperature stress. Furthermore, if no deaths occur within a consecutive four-hour period at 32 °C, the temperature is further increased by 1 °C, and this process is repeated until all individuals have perished. This approach enables precise acquisition of the survival duration for each individual, ensuring an accurate collection of heat tolerance phenotypes.

High-density genetic linkage maps are indispensable tools that are widely used in the genetic breeding of economically important species; these maps support genome assembly, fine-scale QTL mapping, and positional cloning of target genes [22]. A pivotal prerequisite for the construction of a high-density linkage map is the availability of a large number of accurately genotyped genetic markers, which can be challenging for non-model organisms. The advent of GBS approaches has prioritized SNP markers for map construction because of their abundance, widespread availability, and ease of genotyping [13]. The GBS-based linkage map for *L. polyactis* previously published by Liu et al. [36] represents the first high-density genetic map for this species and facilitated QTL mapping for growth traits. In the present study, QTL analysis for heat tolerance in *L. polyactis* was conducted using survival duration and survival status under heat stress as metrics; heat tolerance was treated as a continuous quantitative trait, and 3237 bin markers were employed. Three genome-wide significant QTLs for survival duration and one for survival status were identified. Furthermore, 616 genes within the QTL interval for survival duration and 375 genes within the QTL interval for survival status were also identified.

GWAS is an efficient method for exploring novel loci responsible for trait variation in animals [37]. Numerous studies have utilized GWAS to examine heat tolerance in species such as Atlantic salmon (*Salmo salar*) [25], grass carp (*Ctenopharyngodon idella*) [38], rainbow trout (*Oncorhynchus mykiss*) [39], dwarf surf clam (*Mulinia lateralis*) [40], and Japanese flounder (*Paralichthys olivaceus*) [41]. A notable challenge of QTL mapping is the occurrence of false positive associations stemming from family relatedness [42]. Therefore, in this study, GWAS was conducted as a complementary colocalization method. Following GWAS, 27 significantly associated SNPs for survival duration and one for survival status were discovered, leading to the identification of a total of 66 genes related to heat tolerance. By comparing the candidate genes obtained from QTL mapping and GWAS, 56 overlapping genes were found. Functional enrichment analysis showed that most of these genes are associated with biological processes, the immune system, reproduction, lipid metabolism, and the endocrine system, offering insights into the genetic basis underlying heat tolerance in *L. polyactis*. Notably, six genes were significantly differentially expressed, of which *smpd5*, *polr3d*, *rab11fip2*, and *gfpt1* were upregulated under heat stress, while *gpat4* and *grk5* were downregulated. These six genes are intricately involved in the metabolic processes of fish in response to heat stress; they substantially affect lipid metabolism, amino acid metabolism, carbohydrate metabolism, nucleotide metabolism, transcription, and the immune system.

Among these candidate genes, *smpd5* plays a crucial role in sphingomyelin metabolism, a sphingolipid that is integral to cell membranes [43]. It catalyzes the hydrolysis of sphingomyelin to generate ceramide [44], a key signaling molecule involved in various cellular processes, including apoptosis [45] and the stress response [46]. In this study, *smpd5* was significantly upregulated by approximately 1.64-fold under heat stress, suggesting its potential role in the response to heat stress. Furthermore, the sphingolipid metabolism pathway, encompassing *smpd5*, was enriched, indicating that the smpd5-mediated modulation of sphingolipid metabolism may contribute to overall cellular adaptation to elevated temperatures.

*Rab11fip2* encodes the Rab11-FIP2 protein, which is characterized by a conserved carboxyl-terminal Rab11-binding domain and belongs to the Rab11-FIPs family [47,48]. The Rab11 family plays critical roles in membrane identity, vesicle budding, uncoating, cytokinesis, motility, and fusion by recruiting specialized effector proteins [49,50,51]. Rab11-FIP2, which was initially identified as a Rab11A-binding protein in a yeast two-hybrid screen [47], interacts with Myosin Vb and participates in Rab11-mediated recycling pathways [52]. Research increasingly indicates that Rab11-FIP2 plays an important role in mediating early endocytic recycling [53]. In the present study, *rab11fip2* was significantly upregulated by approximately 4.82-fold under heat stress, implying that an expression is modulated in response to heat stress, thereby suggesting a potential role in the response to heat stress.

*Polr3d* encodes the RPC4 subunit of RNA polymerase III, which is implicated in both the initiation and termination of transcription [54]. In this study, *polr3d* enriched the cytosolic DNA-sensing pathway. DNA-directed RNA polymerase (Polr) complexes catalyze RNA synthesis using DNA as a template. RNA polymerase-associated proteins (RPAPs) can influence the biogenesis of RNA polymerase and play important roles in the immune-related cytosolic DNA-sensing pathway [55,56]. In the study, one DNA-dependent RNA polymerase III subunit (*polr3d*) was upregulated after 6 h of heat stress. This finding further emphasizes that the biogenesis of RNA polymerase may be influenced by heat stress and may play a key role in the heat stress response, as POLR3D has been associated with leukodystrophy phenotypes and genetic diseases [57].

Glutamine fructose-6-phosphate transaminase 1 (GFPT1), encoded by *Gfpt1*, is the first and rate-limiting enzyme of the hexosamine biosynthesis pathway, which is involved in glucose metabolism and plays a critical role in energy regulation [58]. Gfpt1 is a mediator of the unfolded protein response (UPR), as its absence compromises the UPR, leading to reduced autophagy and increased apoptosis [59]. Under the UPR, Xbp1-s induces the expression of *gfpt1*, which increases the generation of UDP-GlcNAc and enhances proper protein glycosylation to suppress cell death [60]. Thus, Gfpt1 is essential for maintaining protein homeostasis under the UPR and mitigating endoplasmic reticulum stress [59]. It has been reported that endoplasmic reticulum stress and the subsequent UPR are more pronounced under heat stress [61]. In the present study, *gfpt1* was significantly upregulated by more than 11-fold under heat stress, suggesting that its expression was upregulated in response to heat stress, thereby indicating its potential role in the heat stress response.

Glycerol-3-phosphate acyltransferase 4 (*Gpat4*) belongs to the glycerol-3-phosphate acyltransferase (GPAT) family. Absence of GPAT4 leads to subdermal lipodystrophy, a significant reduction in triacylglycerol content in adipose tissue and liver [62], and resistance to diet-induced obesity [63]. Therefore, GPAT4 plays an indispensable role in regulating adipogenesis in vivo by facilitating lipid synthesis [64], which is essential for initiating glycerolipid biosynthesis and the stress response [65]. GPAT4 is an important enzyme involved in lipid biosynthesis and signal transduction. In the present study, the expression of *gpat4* was significantly reduced in response to heat stress, indicating that the fish body reduces lipid synthesis by downregulating *gpat4* expression.

*Grk5* encodes a protein that belongs to the G protein-coupled receptor kinase (GRK) family of serine–threonine protein kinases, which recognize and phosphorylate agonist-activated G protein-coupled receptors to terminate signaling. GRK5 is localized within the nucleus and moderates functions such as DNA transcription in addition to its presence in the plasma membrane [66]. The desensitization process also involves β-arrestin binding and G protein-coupled receptor internalization [67,68,69]. Several studies have reported that *grk5* positively regulates adipogenesis and promotes lipid storage in adipocytes [70]. Wang et al. [71] found that the decreased adipose tissue concentration observed in *grk5* knockdown mice is the result of increased lipolysis. In the current study, the expression of *grk5* was also significantly reduced under heat stress, indicating that fish respond to heat stress by downregulating relevant genes to inhibit adipogenesis.

Finally, it is important to note that these heat tolerance-related genes were validated by examining expression levels after 6 h of heat stress. Gene expression levels were not evaluated at other time points after the heat stress, and therefore, certain key genes may have been overlooked in the analysis. Future research should include analyses of gene expression levels at different time points in relation to the heat stress signal, particularly for genes that did not show significant differences at 6 h. Such analyses can further elucidate the molecular mechanisms underlying heat tolerance in *L. polyactis*. Additionally, future evaluations under long-term heat stress may unlock a comprehensive understanding of the genetic mechanisms triggered in *L. polyactis* in response to heat stress.

## 4. Materials and Methods

### 4.1. Fish Full-Sib Family Production and Sample Collection

The F1 full-sib family of *L. polyactis* was established at Xiangshan Ganwan Aquatic Seed Co., Ltd., Ningbo, Zhejiang Province, China, in April 2022, using an artificial insemination method as described by Liu et al. [7]. Specifically, the male individual was selected from a cultured population, whereas the female was sourced from wild stocks caught in Xiangshan Harbour. During the process of family production, four families were successfully created through exclusive mating of a single male and female fish in each case. The families were subsequently reared in a tank with a volume of 2 m^3^, closely following the rearing protocol described by Liu et al. [72]. After approximately seven months of cultivation, 120 offspring from one of the F1 full-sib families were randomly selected for a high-temperature stress experiment conducted in a 2 m^3^ tank. The experiment commenced with an initial water temperature of (15 ± 0.4) °C, which was systematically increased by 1 °C every 4 h until a stable temperature of (32 ± 0.2) °C was attained, facilitated by a precision temperature controller. Throughout the duration of the experiment, fish were closely monitored for any changes in their response. Fish were considered deceased when they were observed either lying motionless on the bottom of the tank or floating unbalanced on the water surface, showing no response even when touched. If no mortalities were recorded consecutively for 4 h at the predetermined temperature setting, the temperature was increased by an additional 1 °C. This incremental process was iteratively conducted until all experimental fish had succumbed.

Immediately upon their death, fish were collected and weighed, and the precise times of their demise under heat stress were accurately recorded. The survival duration, measured in minutes, was calculated using the following formula: Tn (time of fish death) − T0 (time to first fish death temperature), serving as a crucial trait value for evaluating the heat tolerance of fish in this study. Furthermore, tail fin tissues from each fish were sampled and preserved in absolute ethanol for subsequent extraction of genomic DNA.

### 4.2. Genotyping-by-Sequencing Library Construction and Sequencing

Genomic DNA was extracted from all 120 offspring and their two parents using the TIANGEN Marine Animal DNA Extraction Kit (Beijing, China), strictly following the manufacturer’s protocol. The concentration of the extracted DNA was determined using a NanoDrop 2000 spectrophotometer (Thermo Scientific, Waltham, MA, USA), while the quality of each sample was assessed through electrophoresis on a 1% agarose gel. Subsequently, high-quality DNA from all 122 individuals was used to construct GBS libraries, employing a modified two-enzyme protocol derived from the standard Genotyping-by-sequencing (GBS) methodology [73,74]. In brief, genomic DNA from each sample was double digested with NlaIII and EcoRI restriction enzymes (New England Biolabs, Ipswich, MA, USA), followed by purification using the Qiagen MinElute Reaction Cleanup Kit (Qiagen, Venlo, The Netherlands). Digested products were then mixed with 25 pmol of A1 and A2 adapters per well to ligate adapters to both ends of the DNA fragments. The libraries were then pooled and size-selected (300–400 bp) using a 1% agarose gel and further purified using a PCR purification kit (New England Biolabs). Amplification was performed for 12 cycles using Phusion DNA polymerase (New England Biolabs). The average fragment size of the amplified libraries was assessed on a Bioanalyzer 2100 (Agilent, Santa Clara, CA, USA) using a DNA1000 chip, following a second round of column cleaning. Library quantification was conducted using PicoGreen dye (Invitrogen, Carlsbad, CA, USA). The pooled libraries were adjusted to a final concentration of 10 nmol and sequenced using a PE150 configuration on the HiSeq X10 sequencing platform (Illumina, San Diego, CA, USA).

### 4.3. Single-Nucleotide Polymorphism Genotyping

Raw reads were processed using FASTP to obtain high-quality clean reads, adhering to the following criteria: (1) reads with ≥10% unidentified nucleotides were removed; (2) reads with >50% bases having Phred quality scores of ≤20 were removed; and (3) reads aligned to the barcode adapter were also removed. Following quality control, clean reads from each sample were aligned to the reference genome (GCA_040670005.1) downloaded from the National Center for Biotechnology Information (NCBI) using Burrows-Wheeler Aligner software (v0.7.17) [75] with default settings. SNPs were detected and filtered using GATK’s Variant Filtration tool, applying thresholds (QD < 2.0, FS > 60.0, MQ < 40.0, and GQ < 20) to exclude low-quality and error-prone variants. ANNOVAR (v2017–07-17) [76] was employed to annotate and localize the identified SNPs. SNP calling was performed separately for parents and offspring. A polymorphic locus was defined as a site where at least one of the two parents was heterozygous. For subsequent analysis, SNPs present in >90% of the mapping family members were retained, with a minor allele frequency > 5%, a heterozygosity ratio of <75%, and significant segregation (*p* < 0.001). Samples with genotype deletion rates of >30% or heterozygosity rates of >20% were excluded.

### 4.4. Construction and Evaluation of the Genetic Linkage Map

In this study, linkage analysis was conducted using a pseudo-test cross strategy. SNPs of specific segregation patterns, namely lm × ll (exhibiting 1:1 segregation exclusively in the female parent), nn × np (displaying 1:1 segregation only in the male parent), and hk × hk (showing 1:2:1 segregation in both parents), were selected to construct sex-specific and average genetic maps. Initially, SNPs were assigned to linkage groups based on their physical locations in the reference genome; markers not located on chromosomes were removed. The OrderMarkers2 module of Lep-MAP3 software [77] was utilized to calculate both marker distances and map distances for each chromosome. Subsequently, all genetic linkage maps were visualized using the R LinkageMapView package (v2.1.2).

### 4.5. Fine-Mapping of Quantitative Trait Loci for Heat Tolerance Traits

Bin markers can consolidate a large amount of SNP information, thereby reducing the workload of analysis. QTL mapping using high-density maps typically relies on bin markers to reduce computational time and enhance accuracy. Bin markers were identified using the criteria reported by Chen et al. [78]. The genetic positions of bin markers were determined using the genetic coordinates derived from all previously mentioned SNPs. Subsequently, based on the bin map, QTL analysis was performed to identify QTLs associated with phenotypic variations using the interval mapping algorithm of R/QTL software (v1.50) [79]. During the mapping process, the step size was set to 1 cM, while default values were retained for other parameters.

The likelihood of odd (LOD) thresholds for determining significant QTLs was established through permutation tests, employing 1000 replicates, with a confidence interval of 95%. QTLs with LOD scores exceeding the chromosome-wide LOD threshold were considered significant. In cases where the distance between QTL peaks was less than 10 cM, associated QTLs were combined. A 1.5-LOD support interval was used as the confidence interval for QTLs. However, if the peak extended 10 cM in one direction and the LOD value did not decrease by 1.5, the range within 10 cM on both sides of the peak was defined as the QTL boundary. Genes located within the QTL confidence interval were extracted and identified using information from the reference genome and the annotation file of *L. polyactis*.

### 4.6. Genome-Wide Association Studies

Supplementary to QTL analysis, SNP genotyping data from the 120 offspring individuals were utilized in the GWAS. Initially, samples with abnormal heterosome variants, a missing variant rate greater than 0.2, and high heterozygosity exceeding the threshold of mean + 3 × SD were filtered out as outliers. Additionally, SNPs with more than two alleles were removed using PLINK2 software (version alpha 2.3) [80]. The remaining SNPs were then subjected to GWAS analysis employing GEMMA software (v0.98.1) [81] with a general linear model: y = Xα + e. In the model, y represents the phenotypic value; X is the genotype matrix; α is the vector of genotypic effects; e is the residual vector.

SNPs significantly associated with heat tolerance were identified based on the genomic significance threshold calculated as 0.05 divided by the number of SNPs [82], which represents values corrected by the Bonferroni method. Manhattan plots and Q-Q plots were generated from the GWAS results using the R package CMplot. Candidate genes located within a 50 kb region upstream or downstream of significantly associated markers were identified. A comparative analysis was conducted between these candidate genes and the QTL results, with a focus on genes related to heat stress.

### 4.7. Gene Annotation and Enrichment Analysis

Coexisting candidate genes, identified through the integration of QTL and GWAS analyses, were annotated and subsequently subjected to Gene Ontology (GO) and Kyoto Encyclopedia of Genes and Genomes (KEGG) enrichment analyses using the R package clusterProfiler. Enrichment for terms within the three categories of molecular function, biological process, and cellular component was evaluated using the GO database (http://www.geneontology.org/, accessed on 8 June 2024). Additionally, pathway enrichment analysis was conducted to identify metabolic pathways or signal transduction pathways that were significantly enriched among candidate genes obtained from both QTLs and GWAS. The calculated *p*-values were adjusted using the false discovery rate correction method, with a false discovery rate significance threshold of ≤0.05.

### 4.8. Identification of Candidate High-Temperature Response Genes

To further validate the anticipated responses of candidate genes to high temperatures, qPCR was employed to assess the mRNA expression profiles of genes related to heat tolerance. Healthy fish were subjected to treatments in seawater maintained at 32 °C (Test) or 20 °C (Control). Liver tissue samples were collected from five fish per group, 6 h post-treatment, and immediately frozen in liquid nitrogen for preservation. Total RNA was extracted using the TRIzol™ Reagent (Thermo Scientific), followed by reverse transcription into the first strand of cDNA using the PrimeScript™ II 1st Strand cDNA Synthesis Kit (TaKaRa, Tokyo, Japan).

qPCR analysis was performed using the primers detailed in Table 5. The amplification program followed the manufacturer’s instructions: 40 cycles comprising an initial denaturation at 95 °C for 5 s, annealing at 55 °C for 30 s, and extension at 72 °C for 25 s. A melting curve step was incorporated at the conclusion of the qPCR program to verify the specificity of amplification. β-actin served as a reference gene, and the relative expression levels of candidate genes were quantified using the 2^−ΔΔCT^ method [83].

## 5. Conclusions

This study presents a genetic linkage map with 3237 bin markers spanning 24 linkage groups and totaling 1900.84 centimorgans in *L. polyactis*, using genotyping-by-sequencing of a full-sib family comprising 120 progenies and their two parents. QTL mapping identified four QTLs associated with heat tolerance, which encompassed 648 genes within the QTL intervals. The GWAS analysis further disclosed 76 candidate genes related to heat tolerance, 56 of which overlapped with QTL results. Enrichment analysis showed that these genes are involved in immune response, development, lipid metabolism, and endocrine regulation. qPCR validated significant upregulation of four genes and downregulation of two after 6 h of heat stress. These findings demonstrate their responsiveness to elevated high temperatures. This meta-analysis of QTL mapping and GWAS enhances understanding of heat tolerance genetics in *L. polyactis* and provides tools for improving this trait in cultured fish.

## Figures and Tables

**Table 1:** Summary statistics of sex-averaged linkage and bin map.

Linkage Group	Sex-Averaged Linkage Map			Sex-Averaged Bin Map		
	Marker Number	Genetic Length (cM)	Average Interval (cM)	Marker Number	Genetic Length (cM)	Average Interval (cM)
LG01	191,319	76.13	0.0004	134	76.13	0.57
LG02	183,971	71.79	0.0004	130	71.79	0.55
LG03	207,831	110.40	0.0005	179	110.40	0.62
LG04	186,542	79.89	0.0004	123	79.89	0.65
LG05	189,061	103.93	0.0005	171	103.93	0.61
LG06	165,987	87.01	0.0005	133	87.01	0.65
LG07	189,038	72.12	0.0004	119	72.12	0.61
LG08	187,869	78.80	0.0004	123	78.80	0.64
LG09	137,628	71.49	0.0005	134	71.49	0.53
LG10	166,695	77.68	0.0005	145	77.68	0.54
LG11	197,732	74.37	0.0004	129	74.37	0.58
LG12	167,506	71.77	0.0004	142	71.77	0.51
LG13	155,010	73.53	0.0005	125	73.53	0.59
LG14	119,345	57.10	0.0005	129	57.10	0.44
LG15	214,226	72.26	0.0003	129	72.26	0.56
LG16	123,888	62.96	0.0005	125	62.96	0.50
LG17	167,223	103.65	0.0006	151	103.65	0.69
LG18	156,600	68.39	0.0004	142	68.39	0.48
LG19	183,256	63.82	0.0003	134	63.82	0.48
LG20	144,087	102.22	0.0007	127	102.22	0.81
LG21	167,864	66.05	0.0004	110	66.05	0.60
LG22	151,660	79.52	0.0005	137	79.52	0.58
LG23	129,791	80.01	0.0006	130	80.01	0.62
LG24	74,200	95.98	0.0013	136	95.98	0.71
Total	3,958,329	1900.84	0.0005	3237	1900.84	0.59

**Table 2:** Summary statistics of the candidate quantitative trait loci for the two heat tolerance traits in *L. polyactis*.

Traits	Chr	Location (cM)	Number of SNP	Peak Marker	Peak.LOD	PVE (%)	Genes
Survival duration	Chr8	52.13–62.55	6	Chr8_16044431	5.85	10.13	343
	Chr14	40.43–45.85	10	Chr14_24682001	6.01	13.08	84
	Chr24	57.96–72.17	2	Chr24_16888520	5.00	6.98	189
Survival status	Chr8	50.88–63.80	2	Chr8_16044431	5.02	17.52	375

**Table 3:** Candidate genes related to *L. polyactis* resistance.

Trait	Threshold *p* Value	Chr	SNP Number	Gene Number
Survival duration	7.77 × 10^−9^	Chr8	2	19
		Chr14	25	47
Survival status		Chr8	1	10

**Table 4:** Information on genes related to heat tolerance examined by qPCR.

Markers	Genes	Description
Chr8_14197694	*pyy*	peptide YY-like
	*smpd5*	Sphingomyelin phosphodiesterase 5
	*ccdc12*	coiled-coil domain-containing protein 12
Chr14_24682001	*tcf7l1a*	transcription factor 7-like 1-A isoform X2
	*gpat4*	glycerol-3-phosphate acyltransferase 4
	*ppp1r3c*	protein phosphatase 1 regulatory subunit 3C-like
	*polr3d*	DNA-directed RNA polymerase III subunit RPC4
Chr14_24831514	*INPP5l*	inositol polyphosphate 5-phosphatase A
	*sorbs3*	sorbin and SH3 domain-containing protein 2 isoform X5
Chr14_24852088	*rab11fip2*	rab11 family-interacting protein 2
	*gfpt1*	glutamine-fructose-6-phosphate aminotransferase
Chr14_25191367	*grk5*	G protein-coupled receptor kinase 5 like
Chr14_25303065	*piwil1*	Piwi-like protein 1
	*fzd10*	frizzled-10

**Table 5:** Primer sequences used for qRT-PCR verification.

Genes	Forward Sequence (5′–3′)	Reverse Sequence (5′–3′)
*ppp1r3c*	TCTGCAGGATTTGGGAAGCA	ATGGCCTGTTCGTTGACACT
*pyy*	AACGGCAAGAAAACAGACGA	GGCTTGGCTGGATATGCGT
*smpd5*	ATAAACAGCCCGACGAGGAC	TACCAATGACCCACGGCTTC
*gpat4*	GTATCCTGCTCGGCATCTCC	CATGTAAAGACGCCGGATGC
*try3*	CTGAATGCCCCCATCCTGAG	CTGATTGTTGCACACCACGG
*sorbs3*	GGTGTTGGACTACGGGGAAG	CGATCACCTCGCCTTTACGA
*tcf7l1a*	CACCACCACTTCTCCCTAGC	GATTGGCCGGGTGAGGATAG
*fzd10*	TGGGCTACCTCATCCGACTT	AGCCAGAAACCATGTGAGGG
*inpp5a*	CTGCAACTCCAGTCCTTCCA	ACCAGACTTTGCGTGTCCAG
*ccdc12*	AGGCAGCTAATCCAGAACCC	TCTCCAGTTTCTTCGCCACA
*rab11fib2*	ACAGAGCCGTTTGTACGGAG	TATTCTGAGCACTGACCGGC
*grk5*	TCAAGAGACTGGAGGCTGGA	TGGGATGGAAACACTGCCTG
*gfpt1*	GAACACTCCCGTCTTCCGAG	AGCGCTCCTCTCTCCTTACA
*piwil1*	CCCAGAAGATCCGAGCTGAC	TGAATCTGTTGACGCCTCCC
*hspa8*	GGACGAGTACGATCACCAGC	ATACCTCCTGGCATACCCCC
*polr3d*	CCAGTGAAAACGGAGGTCCA	ACCAGCATCTTTCCCACGAG
*β-actin*	CTCTGTCTGGATCGGAGGCT	GCTGAAGTTGTTGGGTGTTTG

## Data Availability

This whole-genome shotgun project of *L. polyactis* has been deposited in GenBank with accession number GCA_040670005.1.

## Supplementary Materials

The following supporting information can be downloaded at: https://www.mdpi.com/article/10.3390/ijms26041638/s1.

## References

[1] Bonfils C.J.W., Santer B.D., Fyfe J.C., Marvel K., Phillips T.J., Zimmerman S.R.H. (2020). Human influence on joint changes in temperature, rainfall and continental aridity. Nat. Clim. Chang..

[2] Brander K.M. (2007). Global fish production and climate change. Proc. Natl. Acad. Sci. USA.

[3] Pan L., Zhang L., Zhang Y., Xie S., Qin Q. (2018). Effects of acute high temperature stress on the growth, immune response, and antioxidant capacity of juvenile grass carp (*Ctenopharyngodon idella*). Aquac. Res..

[4] Li M., Chen L., Zeng C., Qin J.G., Chen H. (2017). Effects of high temperature stress on oxidative stress, immune response and heat shock protein gene expression in fish: A review. Aquac. Res..

[5] Xiong Y., Yang J., Jiang T., Liu H.B., Zhong X.M., Tang J.H. (2017). Early life history of the small yellow croaker (*Larimichthys polyactis*) in sandy ridges of the South Yellow Sea. Mar. Biol. Res..

[6] Kim S., Lim J., Lee K., Park S. (2016). Effect of twine thickness on size-selectivity of driftnet for the yellow croaker *Larimichthys polyactis* in southwestern Sea of Korea. Chin. J. Oceanol. Limnol..

[7] Liu F., Liu Y., Chu T., Luo B., Zhan W., Chen R. (2019). Interspecific hybridization and genetic characterization of *Larimichthys polyactis* (♀) and *L. crocea* (♂). Aquac. Int..

[8] Sukhan Z.P., Cho Y., Hossen S., Yang S.W., Hwang N.Y., Lee W.K., Kho K.H. (2022). Functional characterization of three GnRH isoforms in small yellow croaker *Larimichthys polyactis* maintained in captivity: Special emphasis on reproductive dysfunction. Biology.

[9] Zhou Q., Wang J., Li J., Chen Z., Wang N., Li M., Wang L., Si Y., Lu S., Cui Z. (2024). Decoding the fish genome opens a new era in important trait research and molecular breeding in China. Sci. China Life Sci..

[10] Everett M.V., Seeb J.E. (2014). Detection and mapping of QTL for temperature tolerance and body size in Chinook salmon (*Oncorhynchus tshawytscha*) using genotyping by sequencing. Evol. Appl..

[11] Ma A., Huang Z., Wang X.A., Xu Y., Guo X. (2021). Identification of quantitative trait loci associated with upper temperature tolerance in turbot, *Scophthalmus maximus*. Sci. Rep..

[12] Lagarde H., Lallias D., Patrice P., Dehaullon A., Prchal M., François Y., D’Ambrosio J., Segret E., Acin-Perez A., Cachelou F. (2023). Genetic architecture of acute hyperthermia resistance in juvenile rainbow trout (*Oncorhynchus mykiss*) and genetic correlations with production traits. Genet. Sel. Evol..

[13] Seeb J.E., Carvalho G., Hauser L., Naish K., Roberts S., Seeb L.W. (2011). Single nucleotide polymorphism (SNP) discovery and applications of SNP genotyping in nonmodel organisms. Mol. Ecol. Resour..

[14] Lee B.Y., Kim M.S., Choi B.S., Nagano A.J., Au D.W.T., Wu R.S.S., Takehana Y., Lee J.S. (2019). Construction of high-resolution RAD-seq based linkage map, anchoring reference genome, and QTL mapping of the sex chromosome in the marine Medaka Oryzias melastigma. G3.

[15] Chen G., Zhou Y., Yu X., Wang J., Luo W., Pang M., Tong J. (2022). Genome-wide association study reveals SNPs and candidate genes related to growth and body shape in Bighead Carp (*Hypophthalmichthys nobilis*). Mar. Biotechnol..

[16] Zhang M., Ma X., Wang Z., Han Y., Jia Z., Chen D., Xu Y., Qiao Z., Jiang X., Wang L. (2025). Genome-wide association analysis study on host resistance against the *Aeromonas veronii* of largemouth bass Micropterus salmoides. Fish Shellfish Immunol..

[17] Zhao Y., Wang Z., Chen L. (2023). Genome-wide association study identifies novel loci associated with heat tolerance in Nile tilapia (*Oreochromis niloticus*). Aquaculture.

[18] Liu T.D., Huang D.D., Chang L.Y., Qiao T.F., Xia J.H. (2025). Identification of a novel QTL on LG16 associated with acute salt tolerance in Red Tilapia (*Oreochromis* spp.) using GWAS. Mar. Biotechnol..

[19] Fu S., Liu J. (2022). Genome-wide association study identified genes associated with ammonia nitrogen tolerance in *Litopenaeus vannamei*. Front Genet..

[20] Yang J., Lee S.H., Goddard M.E., Visscher P.M. (2011). GCTA: A tool for genome-wide complex trait analysis. Am. J. Hum. Genet..

[21] Korte A., Farlow A. (2013). The advantages and limitations of trait analysis with GWAS: A review. Plant Methods.

[22] Shi R.H., Li C.Y., Qi H.G., Liu S., Wang W., Li L., Zhang G.F. (2020). Construction of a high-resolution genetic map of Crassostrea gigas: QTL mapping and GWAS applications revealed candidate genes controlling nutritional traits. Aquaculture.

[23] Sallam A., Eltaher S., Alqudah A.M., Belamkar V., Baenziger P.S. (2022). Combined GWAS and QTL mapping revealed candidate genes and SNP network controlling recovery and tolerance traits associated with drought tolerance in seedling winter wheat. Genomics.

[24] Zhang X., Ren Z., Luo B., Zhong H., Ma P., Zhang H., Hu H., Wang Y., Zhang H., Liu D. (2022). Genetic architecture of maize yield traits dissected by QTL mapping and GWAS in maize. Crop J..

[25] Ma F., Zhao L., Ma R., Wang J., Du L. (2023). FoxO signaling and mitochondria-related apoptosis pathways mediate tsinling lenok trout (*Brachymystax lenok tsinlingensis*) liver injury under high temperature stress. Int. J. Biol. Macromol..

[26] Charo-Karisa H., Rezk M.A., Bovenhuis H., Komen H. (2005). Heritability of cold tolerance in Nile tilapia, *Oreochromis niloticus*, juveniles. Aquaculture.

[27] Baer C.F., Travis J. (2000). Direct and correlated responses to artificial selection on acute thermal stress tolerance in a live bearing fish. Evolution.

[28] Perry G.M.L., Danzmann R.G., Ferguson M.M., Gibson J.P. (2001). Quantitative trait loci for upper thermal tolerance in outbred strains of rainbow trout (*Oncorhynchus mykiss*). Heredity.

[29] Sun X.W., Liang L.Q. (2004). A genetic linkage map of common carp (*Cyprinus carpio* L.) and mapping of a locus associated with cold tolerance. Aquaculture.

[30] Quinn N.L., McGowan C.R., Cooper G.A., Koop B.F., Davidson W.S. (2011). Identification of genes associated with heat tolerance in Arctic charr exposed to acute thermal stress. Physiol. Genom..

[31] Chu T.Q., Liu F., Qin G., Zhan W., Wang M.J., Lou B. (2020). Transcriptome analysis of the *Larimichthys polyactis* under heat and cold stress. Cryobiology.

[32] Liu F., Zhang T., He Y., Zhan W., Xie Q., Lou B. (2023). Integration of transcriptome and proteome analyses reveals the regulation mechanisms of *Larimichthys polyactis* liver exposed to heat stress. Fish Shellfish Immunol..

[33] Peng J.X., He P.P., Wei P.Y., Zhang B., Zhao Y.Z., Li Q.Y., Chen X.L., Peng M., Zeng D.G., Yang C.L. (2018). Proteomic responses under cold stress reveal unique cold tolerance mechanisms in the Pacific White Shrimp (*Litopenaeus vannamei*). Front. Physiol..

[34] Shi W., Hao C., Zhang Y., Cheng J., Zhang Z., Liu J., Yi X., Cheng X., Sun D., Xu Y. (2017). A combined association mapping and linkage analysis of kernel number per spike in common wheat (*Triticum aestivum* L.). Front. Plant Sci..

[35] Ibrahim A.K., Zhang L., Niyitanga S., Afzal M.Z., Qi J. (2020). Principles and approaches of association mapping in plant breeding. Trop. Plant Biol..

[36] Liu F., Zhan W., Xie Q.P., Chen H.L., Lou B., Xu W.T. (2020). A first genetic linage map construction and QTL mapping for growth traits in *Larimichthys polyactis*. Sci. Rep..

[37] Zhang Q., Zhang Q., Jensen J. (2022). Association Studies and Genomic Prediction for Genetic Improvements in Agriculture. Front. Plant Sci..

[38] Jin Y., Zhou T., Geng X., Liu S., Chen A., Yao J., Jiang C., Tan S., Su B., Liu Z. (2017). A genome-wide association study of heat stress-associated SNPs in catfish. Anim. Genet..

[39] Yoshida G.M., Yáñez J.M. (2021). Increased accuracy of genomic predictions for growth under chronic thermal stress in rainbow trout by prioritizing variants from GWAS using imputed sequence data. Evol. Appl..

[40] Wang H., Yang Z., Wang S., Zhao A., Wang H., Liu Z., Sui M., Bao L., Zeng Q., Hu J. (2024). Genome-wide association analysis reveals the genetic basis of thermal tolerance in dwarf surf clam *Mulinia lateralis*. Genomics.

[41] San L.Z., Wang G.X., He Z.W., Liu Y.F., Cao W., Zhang Y.T., Yang Y.C., Han T., Qin Y.W., Yang T.L. (2024). Genome-wide association study for high-temperature tolerance in the Japanese flounder. Animal.

[42] Khan A., Ahmad M., Shani M.Y., Khan M.K.R., Rahimi M., Tan D.K.Y. (2024). Identifying the physiological traits associated with DNA marker using genome wide association in wheat under heat stress. Sci. Rep..

[43] Rizzo M.A., Kolesnick R.N. (2000). Sphingomyelinase and ceramide in cell signaling. J. Biol. Chem..

[44] Yabu T., Shimuzu A., Yamashita M. (2009). A novel mitochondrial sphingomyelinase in zebrafish cells. J. Biol. Chem..

[45] Levade T., Malagarie-Cazenave S., Gouazé V., Ségui B., Tardy C., Betito S., Andrieu-Abadie N., Cuvillier O. (2002). Ceramide in apoptosis: A revisited role. Neurochem. Res..

[46] Hannun Y.A., Luberto C. (2000). Ceramide in the eukaryotic stress response. Trends Cell Biol..

[47] Hales C.M., Griner R., Hobdy-Henderson K.C., Dorn M.C., Hardy D., Kumar R., Navarre J., Chan E.K., Lapierre L.A., Goldenring J.R. (2001). Identification and characterization of a family of Rab11-interacting proteins. J. Biol. Chem..

[48] Prekeris R., Davies J.M., Scheller R.H. (2001). Identification of a novel Rab11/25 binding domain present in Eferin and Rip proteins. J. Biol. Chem..

[49] Schafer J.C., Baetz N.W., Lapierre L.A., McRae R.E., Roland J.T., Goldenring J.R. (2014). Rab11-FIP2 interaction with MYO5B regulates movement of Rab11a-containing recycling vesicles. Traffic.

[50] Welz T., Wellbourne-Wood J., Kerkhoff E. (2014). Orchestration of cell surface proteins by Rab11. Trends Cell Biol..

[51] Finger F.P., White J.G. (2002). Fusion and fission: Membrane trafficking in animal cytokinesis. Cell.

[52] Hales C.M., Vaerman J.P., Goldenring J.R. (2002). Rab11 family interacting protein 2 associates with Myosin Vb and regulates plasma membrane recycling. J. Biol. Chem..

[53] Naslavsky N., Rahajeng J., Sharma M., Jovic M., Caplan S. (2006). Interactions between EHD proteins and Rab11-FIP2: A role for EHD3 in early endosomal transport. Mol. Biol. Cell.

[54] Girbig M., Misiaszek A.D., Vorländer M.K., Lafita A., Grötsch H., Baudin F., Bateman A., Müller C.W. (2021). Cryo-EM structures of human RNA polymerase III in its unbound and transcribing states. Nat. Struct. Mol. Biol..

[55] Bernard G., Chouery E., Putorti M.L., Tétreault M., Takanohashi A., Carosso G., Clément I., Boespflug-Tanguy O., Rodriguez D., Delague V. (2011). Mutations of POLR3A encoding a catalytic subunit of RNA polymerase Pol III cause a recessive hypomyelinating leukodystrophy. Am. J. Hum. Genet..

[56] Choquet K., Pinard M., Yang S., Moir R.D., Poitras C., Dicaire M.J., Sgarioto N., Larivière R., Kleinman C.L., Willis I.M. (2019). The leukodystrophy mutation Polr3b R103H causes homozygote mouse embryonic lethality and impairs RNA polymerase III biogenesis. Mol. Brain.

[57] Watt K.E., Macintosh J., Bernard G., Trainor P.A. (2022). RNA polymerases I and III in development and disease. Semin. Cell Dev. Biol..

[58] Farshadyeganeh P., Nazim M., Zhang R., Ohkawara B., Nakajima K., Rahman M.A., Nasrin F., Ito M., Takeda J.I., Ohe K. (2023). Splicing regulation of GFPT1 muscle-specific isoform and its roles in glucose metabolisms and neuromuscular junction. iScience.

[59] Zhang R., Farshadyeganeh P., Ohkawara B., Nakajima K., Takeda J.I., Ito M., Zhang S., Miyasaka Y., Ohno T., Mori-Yoshimura M. (2024). Muscle-specific lack of Gfpt1 triggers ER stress to alleviate misfolded protein accumulation. Dis. Models Mech..

[60] Wang Z.V., Deng Y., Gao N., Pedrozo Z., Li D.L., Morales C.R., Criollo A., Luo X., Tan W., Jiang N. (2014). Spliced X-box binding protein 1 couples the unfolded protein response to hexosamine biosynthetic pathway. Cell.

[61] Zhao X., Li L., Li C., Liu E., Zhu H., Ling Q. (2022). Heat stress-induced endoplasmic reticulum stress promotes liver apoptosis in largemouth bass (*Micropterus salmoides*). Aquaculture.

[62] Wilfling F., Wang H., Haas J.T., Krahmer N., Gould T.J., Uchida A., Cheng J.X., Graham M., Christiano R., Fröhlich F. (2013). Triacylglycerol synthesis enzymes mediate lipid droplet growth by relocalizing from the ER to lipid droplets. Dev. Cell.

[63] Cao G., Konrad R.J., Li S.D., Hammond C. (2012). Glycerolipid acyltransferases in triglyceride metabolism and energy homeostasis-potential as drug targets. Endocr. Metab. Immune Disord.-Drug Targets.

[64] Shan D., Li J.L., Wu L., Li D., Hurov J., Tobin J.F., Gimeno R.E., Cao J. (2010). GPAT3 and GPAT4 are regulated by insulin-stimulated phosphorylation and play distinct roles in adipogenesis. J. Lipid Res..

[65] Bai Y., Shen Y., Zhang Z., Jia Q., Xu M., Zhang T., Fang H., Yu X., Li L., Liu D. (2021). A GPAT1 Mutation in *Arabidopsis* Enhances Plant Height but Impairs Seed Oil Biosynthesis. Int. J. Mol. Sci..

[66] Sok V., Jacinto A.Z., Peng N., Eldemerdash M., Le L., Tran P.D., Feng L.F., Patel J.R., Gi M., Ammon J.C. (2021). G protein coupled receptor kinase 5 modifies the nucleolar stress response activated by actinomycin D. Biochem. Cell Biol..

[67] Pfleger J., Gresham K., Koch W.J. (2019). G protein-coupled receptor kinases as therapeutic targets in the heart. Nat. Rev. Cardiol..

[68] de Lucia C., Eguchi A., Koch W.J. (2018). New insights in cardiac β-Adrenergic signaling during heart failure and aging. Front. Pharmacol..

[69] Sato P.Y., Chuprun J.K., Schwartz M., Koch W.J. (2015). The evolving impact of G protein-coupled receptor kinases in cardiac health and disease. Physiol. Rev..

[70] Kuppusamy P., Ilavenil S., Hwang I.H., Kim D., Choi K.C. (2021). Ferulic acid stimulates adipocyte-specific secretory proteins to regulate adipose homeostasis in 3T3-L1 adipocytes. Molecules.

[71] Wang F., Wang L., Shen M., Ma L. (2012). GRK5 deficiency decreases diet-induced obesity and adipogenesis. Biochem. Biophys. Res. Commun..

[72] Liu F., Zhan W., Xie Q., Lou B., Han M., Xu W., Tao S.S. (2022). First genetic evaluation of growth traits in *Larimichthys polyactis* to guide the formulation of selective breeding programs. Aquaculture.

[73] Elshire R.J., Glaubitz J.C., Sun Q., Poland J.A., Kawamoto K., Buckler E.S., Mitchell S.E. (2011). A robust, simple genotyping-by-sequencing (GBS) approach for high diversity species. PLoS ONE.

[74] Poland J.A., Brown P.J., Sorrells M.E., Jannink J.L. (2012). Development of high-density genetic maps for barley and wheat using a novel two-enzyme genotyping-by-sequencing approach. PLoS ONE.

[75] Li H., Durbin R. (2009). Fast and accurate short read alignment with Burrows-Wheeler transform. Bioinformatics.

[76] Wang K., Li M., Hakonarson H. (2010). ANNOVAR: Functional annotation of genetic variants from high-throughput sequencing data. Nucleic Acids Res..

[77] Rastas P. (2017). Lep-MAP3: Robust linkage mapping even for low-coverage whole genome sequencing data. Bioinformatics.

[78] Chen S., Tian Y., Li Z., Liu Y., Wang L., Li L., Pang Z., Yang C., Wang Q., Shao G. (2022). Construction of a high-density genetic linkage map and qtl mapping for growth traits in hybrid *Epinephelus fuscoguttatus* (♀) and *Epinephelus tukula* (♂) progeny. Aquaculture.

[79] Arends D., Prins P., Jansen R.C., Broman K.W. (2010). R/qtl: High-throughput multiple QTL mapping. Bioinformatics.

[80] Chang C.C., Chow C.C., Tellier L.C., Vattikuti S., Purcell S.M., Lee J.J. (2015). Second-generation PLINK: Rising to the challenge of larger and richer datasets. Gigascience.

[81] Zhou X., Stephens M. (2012). Genome-wide efficient mixed-model analysis for association studies. Nat. Genet..

[82] Lander E., Kruglyak L. (1995). Genetic dissection of complex traits: Guidelines for interpreting and reporting linkage results. Nat. Genet..

[83] Livak K.J., Schmittgen T.D. (2001). Analysis of relative gene expression data using realtime quantitative PCR and the 2^−ΔΔCT^ method. Methods.

